# Operational Informatics in Cancer Centers: Emerging Trends to Support Cancer Care Delivery and Quality Improvement

**DOI:** 10.1055/s-0046-1816607

**Published:** 2026-11-03

**Authors:** Teja Ganta

**Affiliations:** 1 Tisch Cancer Institute, Division of Hematology and Medical Oncology, Icahn School of Medicine at Mount SinaiNew YorkNYUSA; 2 Digital and Technology Partners, Mount Sinai Health SystemNew YorkNYUSA

**Keywords:** Cancer Care Facilities, Delivery of Health Care, Medical Informatics, Digital Health

## Abstract

**Objectives:**

Cancer centers evolved to meet the growing demands of cancer care, integrating various clinical services within multidisciplinary facilities. The structure of operations greatly influences their ability to address challenges and establish effective systems. This study reviews key informatics developments in health services research and quality improvement, demonstrating how digital health solutions have addressed operational issues in oncology.

**Methods:**

The MEDLINE database and the ASCO Meeting Library were searched to identify peer-reviewed articles and conference abstracts published between 2022 and 2025 that reported outcomes of digital health or informatics interventions that supported oncology clinical services.

**Results:**

Key themes in cancer informatics were summarized. Many implementations focus on influencing oncologist behavior to standardize practices or reduce administrative burdens for resource allocation. Other tools utilize novel technologies for efficient care delivery or managing cancer data to drive clinical programs. Advancements in patient engagement are also evident, bridging the gap between patients and providers. Across all areas of opportunity, a growing body of work aims to incorporate AI tools to further enhance the effectiveness of care programs.

**Conclusions:**

While informatics solutions can be integrated into various aspects of cancer care delivery, significant variability exists in evaluating these programs’ success through different process measures and clinical outcomes. Further efforts are needed to standardize the utility of these tools across disparate health systems.

## 1. Introduction

Cancer centers developed to address the increasing demands and complexities of cancer care, necessitating the integration of various clinical services within multidisciplinary facilities [ 1 ]. The American Society of Clinical Oncology (ASCO) survey of oncology practices demonstrates significant variability in the types of services offered at most locations. Most include access to hematology or oncology providers and medication administration, while others also include more expansive offerings such as psychosocial support, genetic counseling, specialty laboratory and imaging facilities, and clinical research [ 2 ]. Also, many oncology practices now coordinate services across networked sites in a geographic area, stressing the need for standardized workflows regardless of a patient’s point of entry into the system [ 3 ]. Several organizations across Europe and the United States have sought to define and set benchmarks for the key characteristics of a high-performing cancer center [ 1 , 4 ]. Despite many differences in how individual cancer centers may operate, all emphasize patient-centeredness to deliver multidisciplinary, pathway-based care that can standardize complex treatment protocols and patient journeys [ 4 ].

How cancer centers organize their operations significantly influences their ability to address these increasing challenges and develop sustainable, efficient cancer care systems. With the escalating complexity of health systems and the rising digitization of services, it’s clear that the future of the health workforce will need to be adept at incorporating novel technologies into routine practice. Operational informatics aims to integrate health data and information systems for “improving outcomes, increasing quality, and reducing costs by enhancing efficiency, productivity, and the management of resources across all care delivery settings.” [ 5 ]. By optimizing various facets of healthcare delivery, informatics principles can modernize the healthcare workforce and ultimately improve patient experiences and outcomes. The following summarizes key informatics developments in the fields of health services research and quality improvement, highlighting how digital health solutions have been implemented in oncology settings to address operational challenges.

## 2. Methods

The MEDLINE database was searched to identify peer-reviewed articles published between 2022 and 2025 that reported any quantitative or qualitative outcomes of digital health or informatics interventions that supported the conduct of any component of clinical services in oncology practices. Additionally, a targeted review of the American Society of Clinical Oncology (ASCO) Meeting Library in relevant topic tracks—”Technology and Innovation in Cancer Care,” “Care Delivery and Quality Care,” and “Health Services Research”—was conducted to identify conference abstracts of similar scope presented in any ASCO meeting between 2022 and 2025. Articles were reviewed to identify key themes and challenges in cancer operational informatics.

## 3. Results and Discussion

### 3.1 Practice and Administrative Efficiency

Adhering to practice standards is vital to creating a culture of quality and safety in health systems [ 6 ]. This importance is highlighted in cancer care, as many national organizations such as the American Society of Clinical Oncology (ASCO), the Oncology Nursing Society (ONS), and the Commission on Cancer (CoC) aim to develop best practices to measure and improve care processes to minimize variation in cancer care delivery and improve patient outcomes [ 7 ]. Healthcare often falls below standards due to various factors, including a lack of provider knowledge regarding best practices, resource challenges like limited staffing or funding, and inadequate integration of services across disciplines [ 6 ]. Clinical decision support (CDS) systems hold significant potential to enhance the efficiency of cancer care delivery; however, important questions remain about their optimal use—particularly how to integrate them into specialized oncology workflows and whether they truly improve cancer outcomes [ 8 ].

Recent developments in CDS demonstrate its capacity to influence cancer providers’ adherence to practice standards. Many institutions have implemented EHR-assisted clinical pathway programs. For instance, Kaiser Permanente has seamlessly integrated oncology care pathways into the EHR as interactive graphical process diagrams with patient-specific contextual information and an interface to transition to treatment plan order entry directly. Utilization of the tool grew considerably over three years, with over 90% of treating oncologists using the tool to order a cancer treatment protocol [ 9 ]. CDS implementations can also improve the safety of cancer therapy selection. Lam *et al.* [ 10 ] established a pre-chemotherapy order set that prompted clinicians to verify essential laboratory measures linked to an elevated risk of chemotherapy-related adverse events, resulting in a notable increase in the timely ordering of some but not all laboratory tests. The reasons why some laboratory tests are more amenable to intervention than others require further clarification. Genomic laboratory testing is increasingly vital for cancer care as it helps identify molecularly targeted therapy; however, testing rates can be suboptimal due to rapidly evolving guidelines and difficulties in the ordering process [ 11 ]. Le *et al.* [ 11 ] optimized EHR functionality that allowed cancer providers to order genomic testing directly from a decision support interface, queuing the order in the EHR. This process reduced the time required to order necessary testing and led to sustainable long-term increases in guideline adherence rates [ 11 ]. Aside from test ordering, CDS targeting provider behavior can prioritize referrals to other specialty services and improve providers’ clinical documentation practices to adhere to quality metrics [ 12 – 14 ].

Several implementations have explored the use of artificial intelligence (AI) to enhance the capabilities of oncology CDS. Mahuron *et al.* [ 15 ] developed a machine learning (ML) model to predict the risk of complications following cancer surgeries and integrated it into a CDS. When a surgeon submits a surgical case request in the EHR, they receive an alert displaying the patient’s ML-derived risk score. While 75% of providers agreed that the alert was provided at the appropriate time, only 29% reported that it influenced their decision to operate.This highlights the significance of assessing provider acceptance of CDS, which may affect its uptake and clinical impact. Several institutions have aimed to solve the problem of improving advanced care planning rates for patients with advanced cancer by delivering ML-derived mortality risk predictions to clinical teams. When clinicians speak with patients about their understanding of their illness and elicit their values, they can enhance patient quality of life and reduce unnecessary treatments; however, many patients are not given this opportunity due to various factors, including inaccurate clinician prognostication [ 16 ]. Using ML-based mortality risk prediction for discussion planning is feasible and improves end-of-life care quality metrics [ 16 – 19 ]. The implementation at the University of Pennsylvania further studied this by analyzing the effect of removing the CDS after a planned three-year clinical trial, which showed a subsequent decrease in advanced care planning documentation rates. This suggests that sustainable CDS is necessary for enacting long-term practice changes [ 20 ]. A secondary analysis of the cost savings associated with this program measured a cumulative savings of $13 million among patients included in the CDS intervention, showing that in addition to improving practice standards, it may also improve resource allocation in health systems [ 21 ]. So far, the literature on ML-derived mortality risk prediction is the most thorough example of how CDS can be integrated through different workflows to address a similar issue and result in sustainable and significant advancements in cancer care delivery.

Other tools, rather than directly changing provider behavior, aim to improve administrative efficiency and ease the burden on healthcare providers. For example, prior authorizations for cancer medications are a significant administrative burden for providers that can take considerable time away from providing direct patient care. A workflow that leveraged EHR data and the work of cancer registrars to more efficiently process authorization requests led to a decrease in the time a medication authorization request spent pending review and the overall denial rate of medications, correlating to an overall cost savings of approximately $27 million for the health system [ 22 , 23 ]. Admane *et al.* [ 24 ] aimed to optimize the utilization of medical appointments at a cancer center by using an EHR query tool to identify patients who were currently hospitalized and, therefore, not likely to come to upcoming appointments. By replacing these appointments with patients who were on the waitlist, they were able to improve care utilization. Similarly, AI has also been integrated into administrative workflows to enhance efficiency. Ambient listening technology enables automated clinical documentation, saving providers an average of 1.5 hours per week [ 25 ]. An AI-based platform was utilized to improve scheduling at a cancer clinic, demonstrating significant improvements in treatment volume and efficiency, along with notable reductions in treatment delays and the need for extended staff work hours [ 26 ].

### 3.2 Clinical Service Delivery

Patients with cancer often encounter challenges in accessing care due to a shortage of nearby subspecialty providers, the logistical strains of commuting to multiple recurring appointments, or even physical limitations arising from cancer burden or treatment side effects [ 27 ]. Consequently, the healthcare system has investigated innovative ways to deliver clinical services through telehealth, a communication system that connects patients and healthcare providers via a virtual platform [ 28 ]. Telehealth can service diverse needs by integrating with various clinical workflows or focusing on specific health system domains. By extending the provider’s offices into the patient’s home, the goal is for patients to navigate the system more efficiently and access services they might not otherwise have received.

Recent implementations have demonstrated reasonable uptake of telehealth services with minimal safety concerns. Hansen *et al.* [ 29 ] completed a pre- and post-implementation study of telehealth for patients with gastrointestinal cancers and showed that telehealth was associated with increased patient interaction and engagement. The rise in engagement primarily stemmed from a further increase in messaging via phone calls and messages in the EHR. Alexander *et al.* [ 30 ] studied the effect of telehealth implementation on cancer patients’ logistic burden and showed that implementation leads to a decrease in patient’s financial burden and a decrease in loss of productivity due to less travel time. Patient-reported experiences have also demonstrated high satisfaction with telehealth implementations, and many patients rate them as easy to use or would likely recommend the system to others. [ 31 – 33 ]. These findings suggest that patients view telehealth and its associated digital health tools as an overall benefit they would readily embrace if given the opportunity. Whether telehealth services leave gaps in care, though, leading to potential safety concerns, is an essential question. A study of healthcare utilization also focusing on acute care events shows no significant difference in following visits comparing in-person vs. telehealth encounters [ 34 ].

Telehealth isn’t necessarily constrained to core oncologist services, though. Modern cancer centers stress a comprehensive team-based approach to care, incorporating additional support services to help patients through their cancer treatments. The growing infrastructure around telehealth has demonstrated that these services may also feasibly include cancer rehabilitation, genetic counseling, psychosocial therapy, lifestyle management, and support for clinical trial enrollment [ 35 – 39 ]. Patients and providers may be particularly interested in the increased opportunities for obtaining second opinions or specialty consults. Some telehealth implementations specifically focus on a remote second opinion program and report that this can often lead to a change in the management of cancer therapy [ 40 – 42 ]. Palliative care specialties may also be uniquely conducive to implementation. A survey of oncologists and palliative care providers shows that most believe virtual care visits in this space are the way forward, especially when facing staffing shortages or when patients are unwell, making the travel to the hospital too burdensome [ 43 ].

Despite the ease of use of telehealth, certain services may still face backlogs due to a lack of provider availability. An emerging body of research now evaluates the use of AI agents to meet this chasm through chatbots or other AI-assisted programs. The ENGAGE study developed and evaluated an artificial intelligence (AI)-based interactive conversational agent to work directly with patients with cancer undergoing active treatment and provide education before completing genetic testing. Results showed high completion rates, most eventually completing genetic testing and evaluating the tool as “easy to use and informative and convenient.” [ 44 ]. Another study evaluated the use of an AI-based nutrition expert for patients with cancer. The patients included in the study had a high prevalence of nutritional problems but demonstrated high engagement with the tool, with most rating that the program was helpful, improved their quality of life, and improved their symptoms [ 45 ]. It remains unclear whether AI agents can be integrated into key areas of cancer therapeutics or how they might affect the healthcare system, including potential impacts on employment and compensation for healthcare workers.

Whether telehealth enables access to core oncology services or is used to supplement access to additional supportive care, implementation challenges and questions related to equity remain relevant. There are significant disparities in who accesses telehealth services as they are less likely to be Black, female, or married [ 34 ]. A study further evaluated the feasibility of telehealth for Black patients and showed they are significantly less likely compared to White patients to be scheduled for a video visit. Possible reasons are that Black patients were also more likely to report practical constraints, such as issues with using the internet or their devices and not having someone to help them [ 46 ]. It’s clear that as healthcare systems seek to implement telehealth tools widely, they must also consider screening for access barriers and should provide additional support to help patients engage with these tools or continue offering essential services through traditional service delivery methods.

### 3.3 Patient Tracking and Data Capture

To provide more personalized care, oncologists increasingly use advanced assessment tools such as biomarkers or other novel risk predictors to guide treatment selection and determine prognosis. As many clinical or research programs focus on finer populations, not having access to such data may hinder matching patients with appropriate clinical services. Consequently, it is becoming essential to identify and track more clearly defined sub-cohorts of patients and their characteristics beyond traditional tumor classifications. Newer informatics tools aim to optimize cohort selection by identifying patients not easily found through conventional methods or curating additional patient and cancer data elements, ultimately advancing population health programs.

Electronic health records have been utilized mainly to support clinical processes and billing; however, the data gathered during routine clinical care offers valuable insights that can extend beyond their original purpose to drive quality care programs. A significant limitation is that vital information in the EHR can be contained in unstructured data fields such as free text, which is not amenable to standard approaches to querying or abstracting this data for reuse [ 47 , 48 ]. Unstructured data often includes information written in varying ways depending on the individual entering it, leading to significant challenges in interpreting it consistently for analysis; moreover, traditional programmatic query methods are limited to structured data with discrete fields and standardized formats [ 47 ]. For example, a quality program designed to track and reduce cancer medication-related adverse events may be hindered by the inability to measure baseline rates, as many of these events are documented primarily in free-text provider notes. Manela *et al.* [ 49 ] developed an EHR-based reporting tool that systematically identified patients treated with cancer immunotherapy who were later hospitalized for adverse events—achieving higher speed and accuracy than manual chart review. AI-based methods can also be integrated; Block *et al.* [ 50 ] solved a similar challenge by using natural language processing (NLP) to parse patient notes and measure cancer immunotherapy-related adverse event rates within minutes, whereas traditional methods might have taken several weeks. NLP-based tasks have also been used to identify cohorts by extracting information related to patient performance status, cancer stage, and cancer sites of metastases [ 51 , 52 ]. Mortal *et al.* [ 53 ] show how using NLP to extract such patient and tumor characteristics and clinical trial inclusion criteria matched 90% of cancer patients to an appropriate clinical trial, signaling that such technologies can drive programs to improve access and recruitment to treatment programs.

Relying solely on EHR data, though, can be limiting, as it may provide an incomplete view of the patient journey due to its episodic nature—only the data selectively documented in each patient encounter is available [ 48 ]. To accurately address the needs of cancer patients, it is necessary to capture more data, including assessing what happens to the patient outside of healthcare facilities. For example, a program was developed to determine whether patients with breast cancer were adhering to prescribed medications. Not taking medications can significantly impact outcomes and may not be accurately evaluated by traditional methods that rely on the provider specifically eliciting this information from the patient and recording it in the chart. When patients completed the check-in process at a clinic visit, they were asked to complete a questionnaire in the EHR measuring their medication adherence and found that close to a quarter of patients were not adhering as intended [ 54 ]. Measuring electronic patient-reported outcomes (ePRO) such as these can be feasible and advantageous to drive quality programs. Most implementations show that patients rate these questionnaire systems highly and have high completion rates when inputting the required data [ 55 – 58 ]. The rich knowledge gained from these assessment tools has been shown to improve care outcomes meaningfully when appropriately matched with clinical services [ 59 – 62 ]. For example, Daly *et al.* [ 62 ] developed a program to measure ePROs after patients with cancer were discharged from the hospital and return those results to the provider teams in an application that supported secure messages between patients and providers. The program decreased 30-day readmissions, correlating to reduced healthcare spending of approximately $2.3 - $5.8 million annually. The DEFeNDR program targeted patients with cancer who were diagnosed with neutropenic fever, a serious and often deadly complication of chemotherapy, and showed that measuring and responding to ePROs was able to decrease healthcare utilization as patients had a lower risk of hospital admission, ICU utilization, and patient messaging in the EHR [ 61 ].

Even with access to structured or AI-curated data, utilizing it to support operational processes can be challenging, as the information may be poorly organized, erroneous, and scattered across various modules in the EHR or other information systems, making it cumbersome to obtain a broad overview [ 63 ]. Dashboards offer a solution to manage large volumes of data by aggregating and summarizing data from different sources and presenting the knowledge visually in a single interface. These tools can provide timely information to improve patient care by supporting organizational or departmental decision-making [ 63 ]. For example, Sanyal *et al.* developed an operational dashboard displaying key performance metrics to support the cancer center, such as clinical trial accruals, financial statistics, provider productivity, and adherence rates to quality program specifications. The tool was accessible to both clinical and operational staff, and it helped close care process gaps, including reducing the time from provider order entry to imaging scheduling, increasing physician documentation of cancer staging, and boosting the use of tools to measure and track psychosocial distress in patients with cancer [ 64 , 65 ]. Other implementations at cancer centers have also successfully monitored ePROs, health disparities, and parameters of social determinants of health [ 66 – 69 ]. For example, Edmonds *et al.* [ 67 ] developed a dashboard to track delays in initiating breast cancer treatment and found that the tool could identify that delays were more common among minoritized patients, which could help drive care quality programs to fix these care gaps.

### 3.4 Patient Engagement

While innovations like telehealth and ePROs have greatly enhanced the patient experience, care delivery may still be limited when it is dependent on healthcare providers to manage and interpret health data. Previous research in CDS has shown that patient-directed interventions are also feasible, although they are less likely to improve outcomes than those targeting providers. However, questions remain regarding how to effectively adapt these interventions and what factors impact patient acceptance of these tools [ 70 ]. Recent developments in informatics expand on this work to continue developing the patient’s role as an active stakeholder in their care.

Several tools have been studied to assist patients in making decisions about their care or self-managing health issues. Decision aids can come in various formats, such as pamphlets, videos, or interactive web interfaces, to help patients understand their values and the risks and benefits of making treatment decisions [ 71 ]. Implementations for cancer decision-making, such as helping to facilitate the patient choice between surgery or radiation for skin cancer or prioritizing different medications for colon cancer based on outcomes and costs, have shown that patients are largely satisfied with these tools. [ 72 – 74 ]. One implementation, however, examined the impact on patient health knowledge among those awaiting decision-making for prostate cancer management and found no significant improvement from using a decision aid. Whether tailoring these interventions to meet patients’ specific health literacy needs or implementing more interactive formats enhances the effectiveness of decision aids remains an active area of research [ 75 ].

Patient-facing health applications that engage users actively promise to guide patients further. A patient navigator platform designed for individuals with colon cancer was developed and evaluated in a clinical trial to analyze user experience and health outcomes, demonstrating a reduction in anxiety after patients completed their cancer appointments [ 76 ]. Another application, STAMP+CBT, aimed to help patients self-manage cancer-related pain by providing education and guiding cognitive behavioral interventions. Most patients rated the interface highly and reported that the program helped them better manage their symptoms [ 77 ]. Adding AI-based functionality, including chatbots, is also an emerging area of investigation. ChatGPT is a large language model (LLM) chatbot that can summarize large amounts of text, serving as a potential source of educational materials for patients [ 78 ]. Investigators evaluated how effectively it can summarize informed consent forms for clinical trials, demonstrating that it significantly enhanced readability scores, potentially aiding the recruitment of patients facing cognitive or health literacy challenges [ 78 ]. Accuracy is a significant concern for these tools, which can limit their safety for use. When trained to produce content related to financial toxicity, cancer symptoms, or treatment side effects, independent expert validators largely rate the output as well-organized but prone to errors [ 79 , 80 ]. An AI chatbot was developed to message patients with gastrointestinal cancers, helping them adhere to oral chemotherapy medications and manage minor side effects. Patients only engaged with 27% of messages, though, and rated some of the advice as too simplistic to be helpful [ 81 ]. It’s clear that while AI tools can be practically incorporated into patient workflows, it will be vital to involve human experts to continue upholding the quality of health education content.

## 4. Conclusions

Significant variability exists in how informatics solutions are adapted to enhance components of cancer care delivery. The success of these implementations is measured by various indicators, including patient and provider engagement, satisfaction or acceptability, quantitative improvements in process measures, and even measurable impacts on healthcare spending or resource allocation. As most were single-institution projects with minimal use of clinical trial methodologies for evaluation, further efforts are needed to understand how to standardize the utility of these tools across disparate health systems. Across all areas of opportunity, a growing body of work aims to incorporate AI tools to further enhance the effectiveness of care programs. The future healthcare workforce must be skilled at understanding and managing the risks associated with using AI-assisted tools.
